# Epigallocatechine-3-gallate Inhibits the Adipogenesis of Human Mesenchymal Stem Cells via the Regulation of Protein Phosphatase-2A and Myosin Phosphatase

**DOI:** 10.3390/cells11101704

**Published:** 2022-05-20

**Authors:** Bálint Bécsi, Zoltán Kónya, Anita Boratkó, Katalin Kovács, Ferenc Erdődi

**Affiliations:** 1 Department of Medical Chemistry, Faculty of Medicine, University of Debrecen, H-4032 Debrecen, Hungary; bbalint@med.unideb.hu (B.B.); konya.zoltan@med.unideb.hu (Z.K.); boratko@med.unideb.hu (A.B.); kovacs.katalin@med.unideb.hu (K.K.); 2 MTA-DE Cell Biology and Signaling Research Group, Faculty of Medicine, University of Debrecen, H-4032 Debrecen, Hungary

**Keywords:** mesenchymal stem cells, adipogenesis, epigallocatechin-3-gallate (EGCG), 67 kDa laminin receptor (67LR), protein phosphatase-2A (PP2A), myosin phosphatase

## Abstract

Epigallocatechin-3-gallate (EGCG) has widespread effects on adipocyte development. However, the molecular mechanisms of EGCG are not fully understood. We investigate the adipogenic differentiation of human-derived mesenchymal stem cells, including lipid deposition and changes in the expression and phosphorylation of key transcription factors, myosin, protein phosphatase-2A (PP2A), and myosin phosphatase (MP). On day 6 of adipogenic differentiation, EGCG (1–20 µM) suppressed lipid droplet formation, which was counteracted by an EGCG-binding peptide for the 67 kDa laminin receptor (67LR), suggesting that EGCG acts via 67LR. EGCG decreased the phosphorylation of CCAAT-enhancer-binding protein beta via the activation of PP2A in a protein kinase A (PKA)-dependent manner, leading to the partial suppression of peroxisome proliferator-activated receptor gamma (PPARγ) and adiponectin expression. Differentiated cells exhibited a rounded shape, cortical actin filaments, and lipid accumulation. The EGCG treatment induced cell elongation, stress fiber formation, and less lipid accumulation. These effects were accompanied by the degradation of the MP target subunit-1 and increased the phosphorylation of the 20 kDa myosin light chain. Our results suggest that EGCG acts as an agonist of 67LR to inhibit adipogenesis via the activation of PP2A and suppression of MP. These events are coupled with the decreased phosphorylation and expression levels of adipogenic transcription factors and changes in cell shape, culminating in curtailed adipogenesis.

## 1. Introduction

Obesity is a major public health problem affecting at least one-third of the world’s population. Obesity is linked to many diseases, including coronary heart diseases such as hypertension and arteriosclerosis, insulin resistance, diabetes, and metabolic syndrome [1,2,3]. In adulthood, the number of adipocytes is constant and obesity is coupled with an increase in adipocyte size and numbers [4]. However, there is a limited annual turnover of adipocytes (ca 10%/year), requiring new adipocyte development [4]. Therefore, understanding the regulation of adipocyte development and function will facilitate our understanding of adipocyte dysfunction leading to obesity-induced pathologies.

Adipocytes differentiate from mesenchymal stem cells (MSCs) in a multistep process, termed adipogenesis, which is controlled by many intracellular and extracellular factors [5]. Two families of transcription factors, namely peroxisome proliferator-activated receptors (PPARs) and CCAAT-enhancer-binding proteins (C/EBPs), are important determinants of adipogenic differentiation. Upon the induction of the adipogenic differentiation of preadipocytes or MSCs, the expression of C/EBPβ increases followed by PPARγ expression and the synthesis of fatty acids, which are required for the activation of PPARγ. Activated PPARγ enhances the expression of C/EBPα and other adipogenic genes, such as adiponectin, which are necessary for adipocyte development [5,6,7,8].

The activity of C/EBPβ and PPARγ, key transcription factors in adipogenesis, are regulated by post-translational modifications. The phosphorylation and dephosphorylation of C/EBPβ and PPARγ play important roles [9,10] in adipocyte differentiation. The phosphorylation of C/EBPβ at Thr188/235 (mouse/human isoforms) by MAPK and on Ser184 or Thr179 by CDK2 or GSK3β, respectively, increases DNA-binding and facilitates adipogenesis [10]. In contrast, the phosphorylation of PPARγ on Ser82/112 (γ1/γ2 isoforms) by ERK1 or JNK inhibits both its ligand-dependent and -independent transactivation activity [9]. Several types of phosphoserine/threonine (P-Ser/Thr) specific protein phosphatases are involved in the mediation of adipocyte differentiation. The protein phosphatase-1 (PP1) catalytic subunit β/δ (PP1cβ/δ, termed PP1cδ) is essential for the initiation and execution of adipogenesis [11]. The inhibition of protein phosphatase-2A (PP2A) by okadaic acid (OA) blocks adipogenesis at an early phase, but does not influence late phase adipogenesis [12], implying that the presence of active PP2A is required for the initiation of adipogenesis. The PP2A heterotrimer (ABC), including the δ isoform of the B subunit, dephosphorylates C/EBPβ^pThr188^ at the early phase, while PPARγ^pSer112^ and C/EBPα^pSer21^ are dephosphorylated in a PP2A-dependent manner at the later phase of adipogenesis. PPARγ^pSer112^ is also dephosphorylated by protein phosphatase 5 (PP5) [13] or by WIP1 (wild type p53-induced phosphatase 1), also termed as PPM1D, a phosphoserine/threonine-specific type 2C phosphatase [14]. The deletion of PP5 or WIP1 leads to the dysregulation of adipocyte development. Thus, the modulation of distinct protein phosphatases may significantly influence adipogenic differentiation pathways.

Cell shape determines whether MSCs differentiate into adipocytes or osteoblasts [15,16,17]. Adherent, flattened, and spread MSCs undergo osteogenesis, while round non-adherent MSCs differentiate into adipocytes. The rearrangement of the actin cytoskeleton and the formation of actin–myosin stress fibers are important determinants of cell shape and stiffness and are regulated, at least in part, by Rho-family G-proteins and their downstream effectors, including Rho-A activated kinase (ROCK). ROCK activates myosin and facilitates actin–myosin stress fiber formation by phosphorylating the 20 kDa light chain of myosin (MLC20) and, in parallel, by inactivating the myosin phosphatase holoenzyme via the phosphorylation of the myosin phosphatase target subunit-1 (MYPT1) at the Thr696 and/or Thr853 inhibitory sites in the PP1cδ-MYPT1 complex [18,19]. Dephosphorylated myosin favors the round shape and relaxed state of MSCs that promotes adipogenesis; therefore, the requirement of an active myosin phosphatase holoenzyme might be expected for the initiation and execution of adipogenesis.

Chemicals of natural origin, such as catechins in green tea extract, influence adipogenesis, but their effects are controversial: (−)-catechin and its derivatives promote adipocyte differentiation in human bone marrow MSCs through PPARγ transactivation [20], while green tea extracts reduce adipogenesis by decreasing the expression of transcription factors C/EBPα and PPARγ in another experimental setup [21]. Epigallocatechin-3-gallate (EGCG), the major constituent of green tea, attenuated diet-induced obesity in mice by decreasing energy absorption and increasing fat oxidation [22]. EGCG inhibited 3T3-L1 preadipocyte mitogenesis via the 67 kDa laminin receptor (67LR) [23,24] and suppressed differentiation by the EGCG dimer disturbance of 3T3-L1 cell membranes [25]. In bovine bone marrow MSCs, EGCG suppressed lipid deposition through apoptosis [26] and induced free-radical production during adipogenic differentiation [27]. EGCG reduces visceral adiposity [28] and obesity as well as white adipose tissue gain [29].

As described above, EGCG may influence adipogenesis in diverse ways. However, the detailed molecular mechanisms have not been elucidated. EGCG is an agonist of 67LR and signals cells to increase cAMP levels and, consequently, activate protein kinase A (PKA) [30,31]. Previous studies showed that the PKA phosphorylation of the B56δ subunit in the PP2A holoenzyme [32] enhances PP2A activity, resulting in the dephosphorylation of the inhibitory sites in MYPT1, thereby activating MP [31,33]. This EGCG→cAMP→PKA→PP2A→MP activation pathway plays important roles in sensitizing melanoma [31] and leukemic cells [34] to anticancer drugs and in differentiating THP-1 monocytes to macrophages [35]. It remains to be examined if the above EGCG-induced phosphatase activatory pathways may also mediate the adipogenic differentiation of MSCs.

The present study plans to clarify the mechanism of how EGCG influences the adipogenesis of MSCs via interactions with 67LR focusing on downstream signaling pathways, including the possible regulation of PP2A and MP. These phosphatases may mediate the phosphorylation level of adipogenic transcription factors and cytoskeletal proteins important to the changes in cell shape during differentiation; therefore, EGCG-induced changes in their activities could also contribute to the signaling of adipogenesis. Our data show that EGCG suppresses adipogenesis, which coincides with the translocation of 67LR from the cytosol into the cell membrane. The activation of PP2A by EGCG in a PKA-dependent manner results in the decreased phosphorylation and activity of C/EBPβ, which may also contribute to the decreased differentiation of MSCs into adipocytes and lipid droplet formation. Our intriguing finding is that EGCG induces the proteolytic degradation of MYPT1, which in turn increases myosin phosphorylation and the stiffness of cells, contributing to the suppression of adipogenesis.

## 2. Materials and Methods

### 2.1. Materials

L-glutamine, low/high-glucose Dulbecco’s Modified Eagle Medium (DMEM), fetal bovine serum (FBS), penicillin-streptomycin solution, 1,4-diazabicyclo[2.2.2]octane (DABCO), Mowiol 4-88, DAPI (4′,6-diamidino-2-phenylindole), protease inhibitor cocktail (PIC), phosphatase inhibitor cocktail, dexamethasone, indomethacin, 3-isobutyl-1-methylxanthine (IBMX), (−)-epigallocatechin-3-gallate (EGCG), and 9-diethylamino-5-benzo[α]phenoxazinone (Nile Red dye) were obtained from Sigma (St. Louis, MO, USA). All other chemicals were purchased as follows: ProteoJETTM Membrane Protein Extraction Kit, Texas Red Phalloidin, and GeneJET RNA Purification Kit (Thermo Fisher Scientific, Vantaa, Finland); oligo-dT primer and M-MLV reverse transcriptase (Promega Corporation, Madison, WI, USA); inhibitor-2 protein [36] and protein kinase A inhibitor (PKI) (Merck, Darmstadt, Germany); and 67LR^161-180^ peptide, IPCNNKGAHSVGLMWWMLAR (PEPMIC CO., LTD, Suzhou, JS, China). The following antibodies were used: anti-PPARγ (E-8), anti-C/EBPβ (C-19), and anti-PP1c (E9) (Santa Cruz Biotechnology, Santa Cruz, CA, USA); anti-MLC20 and anti-phospho-MLC20 (Cell Signaling Technology, Inc. Beverly, MA, USA); anti-phospho-CEBP-Beta (phospho-Thr188/235) and 67LR (Abcam, Cambridge, UK); anti-phospho-PPARγ (phospho-Ser82/112) (Merck, Darmstadt, Germany); and anti-MYPT1^1–296^ [37], anti-PP1cδ, and monoclonal anti-PP2Ac (Upstate Biotechnology, Lake Placid, NY, USA).

All other chemicals used were of the highest commercially available purity.

### 2.2. Isolation and Phenotyping of Mesenchymal Stem Cells

MSCs were derived from the chorion layer of human placentas (approved by the regional research ethics committee under the license number DEOEC-RKEB-2946-2009). The MSC phenotyping was performed based on the criteria of ISCT (The International Society for Cellular Therapy) [38]. The phenotype of the isolated cells was confirmed, based on the surface antigen pattern using cell staining with appropriate antibodies combined with flow cytometry assays (Becton Dickinson BD FACS Calibur and BD Multiset Software v3.0x for Mac OS X), as described previously [39]. Only cells positive for CD73, CD90, CD105, and CD166 and negative for CD34, CD45, vWF, and HLA-G were used in the experiments.

### 2.3. MSC Culture and Differentiation

MSCs were cultured in low-glucose DMEM supplemented with 10% FBS, 1% L-glutamine, 50 U/mL penicillin, and 50 μg/mL streptomycin. Two days after confluence, the cells were differentiated in an adipogenic medium consisting of high-glucose DMEM supplemented with 0.5 mM IBMX, 1 μM dexamethasone, 200 μM indomethacin, 10 μM insulin, 10% FBS, 1% L-glutamine, 50 U/mL penicillin, and 50 μg/mL streptomycin. Cells were differentiated for 6 days. EGCG was dissolved in sterile water at a concentration of 5 mM and diluted in the adipogenic medium at the concentrations of 1, 5, 10, and 20 μM. The culture medium and adipogenic medium (with or without EGCG) were changed on day 3.

### 2.4. Nile Red Staining and Quantification

MSCs were cultured in 96-well plates or on coverslips. Two days after reaching confluence, the cells were differentiated in an adipogenic medium without or with EGCG at the concentrations of 1, 5, 10, and 20 μM and the absence or presence of 20 μM EGCG, 20 μM 67LR^161-180^ peptide, or 20 μM EGCG plus 20 μM 67LR^161-180^ peptide. After removing the medium, the cells were washed three times with PBS and fixed with 4% paraformaldehyde solution for 30 min on days 0, 3, and 6 of differentiation. The cells were then washed three times with PBS and the lipid droplets were stained with Nile red dye diluted in PBS to 10 μg/mL for 30 min in the dark. The cells were washed three times with PBS and 50 μL of PBS was added to each well. The fluorescence intensity was measured with a fluorescent plate reader (excitation/emission wavelengths: 485/538 nm). Coverslips were washed three times for 10 min in PBS, and DAPI was applied for nuclear staining. The coverslips were then glued on a glass slide with a mounting medium containing Mowiol:Dapco (50:1). MSCs were imaged on a Leica TCS SP8 confocal microscope equipped with Helium/Neon 543, Krypton/Argon 488/568, and Argon 351/364 (UV) laser detectors using 80–110 nm pinholes at 25 °C. Individual random fields were collected using an HC PL APO CS2 63×/1.4 oil DIC immersion objective lens. Images were processed using LAS AF Lite software. The day 6 images were then analyzed by ImageJ software to determine the size and number of lipid droplets. Images were inverted and the image type was set to 8-bit greyscale. After adjusting the threshold, the particle analysis was performed.

### 2.5. Immunofluorescence Staining

MSCs were differentiated on coverslips 2 days after reaching confluence. Samples were collected on days 0, 3, and 6 of differentiation. The medium was removed, cells were washed three times with PBS, fixed with 4% PFA solution for 10 min, washed with PBS again, and permeabilized (0.25% Triton X, 0.1%Tween in PBS) for 30 min. After washing, the cells were blocked with the blocking solution (2% BSA and 0.1% Tween in PBS) and incubated overnight with the primary antibody (67LR, 1:200). Cells were treated for 2 h with the Alexa-488 conjugated secondary antibody (rabbit, 1:200) diluted in blocking solution. DAPI (1:2000) was applied for nuclear staining and Texas Red Phalloidin (1:400) was applied for the staining of actin at the same time as the secondary antibody. Cells were imaged and the colocalization of the 67LR with actin was analyzed on a Leica TCS SP8 confocal microscope with the same settings that were described in the previous section.

### 2.6. Protein Extraction and Western Blotting

MSCs were cultured and differentiated in 6-well plates in the absence or presence of 20 μM EGCG or 20 μM EGCG plus 20 μM protein kinase A inhibitor (PKI). The cells were washed with ice-cold PBS and harvested in 200 μL/well of Ripa buffer (50 mM Tris-HCl pH = 7.4, 150 mM NaCl, 1% TritonX-100, and 0.25% Na-deoxycholate) supplemented with a fresh protease and phosphatase inhibitor cocktail. The cells were lysed in an ice-cold ultrasound water bath for 10 min, followed by short vortexing and centrifugation at 15,700 g for 10 min. The supernatant was boiled for 10 min with 6xSDS sample buffer (60 mM Tris–Cl pH 6.8, 2% SDS, 10% glycerol, and 0.01% bromophenol blue) supplemented with fresh 5% 2-mercaptoethanol. The protein concentration of the lysates was determined with a BCA protein assay at 590 nm on an ELISA reader (Labsystem Multiscan MS). Equal protein amounts of boiled lysates (20 μg) were separated by 10% or 12% SDS-PAGE and transferred to nitrocellulose membranes. Membranes were blocked with 3% BSA in Tris-Buffered Saline (TBS) containing 0.5% Tween-20 (TBST). Membranes were incubated with primary antibodies in appropriate dilutions. The membranes were washed two times with TBST and once with TBS for 10 min, then incubated with horseradish peroxidase-conjugated rabbit or mouse secondary antibody (1:2500). The immunoreactive bands were detected by ECL, imaged, and evaluated with ChemiDocTM Touch Gel Imaging System (Bio-Rad) and densitometric analysis with Image Lab 5.2.1 software. The bands of the proteins of interest were normalized to the loading controls (α-actin or GAPDH) and then related to the signals of day 0 (control).

### 2.7. Assay of Phosphatase Activity

Protein phosphatase and PP2A specific activities were determined as described previously [37]. MSCs were cultured and differentiated in 6-well plates in the absence or presence of EGCG at 1, 5, 10, and 20 μM. On days 0, 3, and 6, cells were washed with PBS and harvested in 200 μL of TBS-EDTA buffer (50 mM Tris/HCl pH = 7.4, 150 mM NaCl, 1 mM EDTA, 50 mM 2-mercaptoethanol, and 0.15% protease inhibitor cocktail), then sonicated with a Branson Sonifer 250 (Output control: 1; Duty Cycle: 10 %; Timer: 30 s) three times for 30 s (30-s break in each cycle). The lysates were centrifuged at 16,100 g for 10 min and 3 × 10 μL of the supernatant was assayed for protein using a BCA assay kit. Supernatants (10 μL in 3-fold final dilution at 0.217–0.446 mg/mL) were preincubated in the absence or presence of 2 µM Inhibitor-2 protein at 30 °C for 1 min, then incubated with 1 µM ^32^P-labeled 20 kDa light chain of gizzard myosin (^32^P-MLC20) substrate for 30 s. The reactions were terminated by the addition of 200 µL 10% trichloroacetic acid and 200 µL 6 mg/mL BSA solutions. The samples were centrifuged for 3 min at 9300 g and 370 μL of supernatant was transferred into scintillation vials. The radioactivity of the released ^32^P_i_ was measured in a Tri-Carb 2800TR scintillation counter instrument. The activity of PP2A was normalized to the protein content of the cell lysates and expressed as relative values compared to the control (phosphatase activity of the lysate at day 0).

### 2.8. Cell Fractionation

Cell fractionation was carried out using ProteoJETTM Membrane Protein Extraction Kit (Thermo Scientific Inc., Waltham, MA, USA) according to the manufacturer’s protocol. Fraction purity was analyzed by Western blot using a CD105 membrane marker antibody.

### 2.9. Reverse Transcription–Polymerase Chain Reaction

MSCs were differentiated in 6-well plates in the absence or presence of 20 μM EGCG. On days 0, 3, and 6 of differentiation, the cells were washed with PBS and stored at –70 °C until use. Total RNA was isolated using GeneJET RNA Purification Kit. The cDNA was synthesized from 2 μg of total RNA using oligo-dT primer and M-MLV reverse transcriptase. For PCR, Phusion^®^ High-Fidelity DNA Polymerase was used. First step denaturation was made at 98 °C for 1 min. It was followed by 20 cycles of denaturation at 98 °C for 5 s, annealing for 5 s at 59 °C for MYPT1 or 48 °C for GAPDH, and extension at 72 °C for 10 s. The final extension was made at 72 °C for 1 min; then, the samples were held at 4 °C. The following primers were used: MYPT1: 5′-CCACAACCCTGACTACAACTAC-3′; 5′-TCTCCTTCTTTCTCCTCTTCTCT-3′; GAPDH: 5′-CATCAATGACCCCTTCAT-3′; 5′-CACAGTCTTCTGGGTG-3′. MYPT1 and GAPDH expressions were determined using 1.2% agarose gel electrophoresis followed by densitometric analysis.

### 2.10. Statistical Analysis

All experiments were carried out at least 3 times and results are expressed as means ± standard deviations. A two-way analysis of variance and Tukey’s multiple comparisons tests were used to compare the multiple treatment conditions with the controls. All analyses were performed using GraphPad Prism 6 version 6.01 software for Windows. A value of *p* ≤ 0.05 was considered statistically significant.

## 3. Results

### 3.1. Effect of EGCG on Lipid Droplet Formation during the Adipogenic Differentiation of MSCs

**Figure 1:**
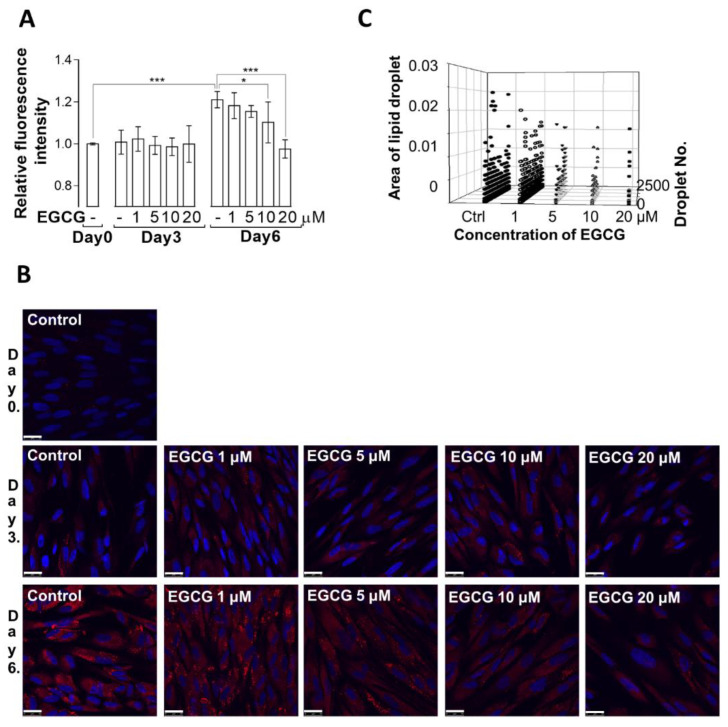
Detection of lipid droplets in MSCs during adipogenic differentiation. MSCs were differentiated in the absence or presence of 1, 5, 10, and 20 μM EGCG, and lipid droplet accumulation was detected in differentiated cells with Nile red dye. (**A**) Lipid droplets in cells in a 96-well plate assay on days 0, 3, and 6 of differentiation using a fluorescent plate reader. Treatments were compared with a two-way ANOVA; * *p* < 0.05, *** *p* < 0.001; n.s. nonsignificant; *n* = 7. (**B**) Lipid droplets (red) stained on coverslips on day 6 and imaged by confocal microscopy. Scale bar: 25 μm. (**C**) The number (right axis) and size (left axis) of lipid droplets as a function of EGCG concentration analyzed by ImageJ.

The literature is somewhat controversial with respect to how catechins and green tea extract, including EGCG, influences adipogenesis [20,21]. Thus, we first studied the effects of a 1–20 µM concentration range of EGCG on lipid droplet formation during the adipogenic differentiation of MSCs. Cells were stained with Nile Red dye and lipid-bound staining was assayed with a fluorescent plate reader (Figure 1A) or on coverslips imaged by confocal microscopy (Figure 1B). As shown in Figure 1A,B, lipid droplet formation increased significantly on day 6 of MSC differentiation and was suppressed by EGCG in a concentration-dependent manner. Day 6 images (Figure 1B) were subjected to ImageJ analysis to assess the effects of EGCG on the size and number of lipid droplets (Figure 1C). The size and number of lipid droplets decreased with increasing concentrations of EGCG on day 6 of adipogenic differentiation.

These data indicate that EGCG suppresses the adipogenic differentiation of human MSCs in accord with previous results in 3T3-L1 pre-adipocytes [24] and bovine bone marrow MSCs [26].

### 3.2. Distinct Localization of 67LR during Differentiation May Influence the Effects of EGCG on Adipogenesis

Many of the cellular effects of EGCG are exerted through its interaction as an agonist with membrane-bound 67LR, thereby inducing various intracellular events, such as increasing cAMP levels and activating PKA [30,31]. Therefore, we assessed the effects of EGCG on adipogenic differentiation via 67LR by using the 67LR^161-180^ peptide representing the EGCG binding region of 67LR, which may counteract the cellular effects exerted by the EGCG–67LR interaction [40].

**Figure 2:**
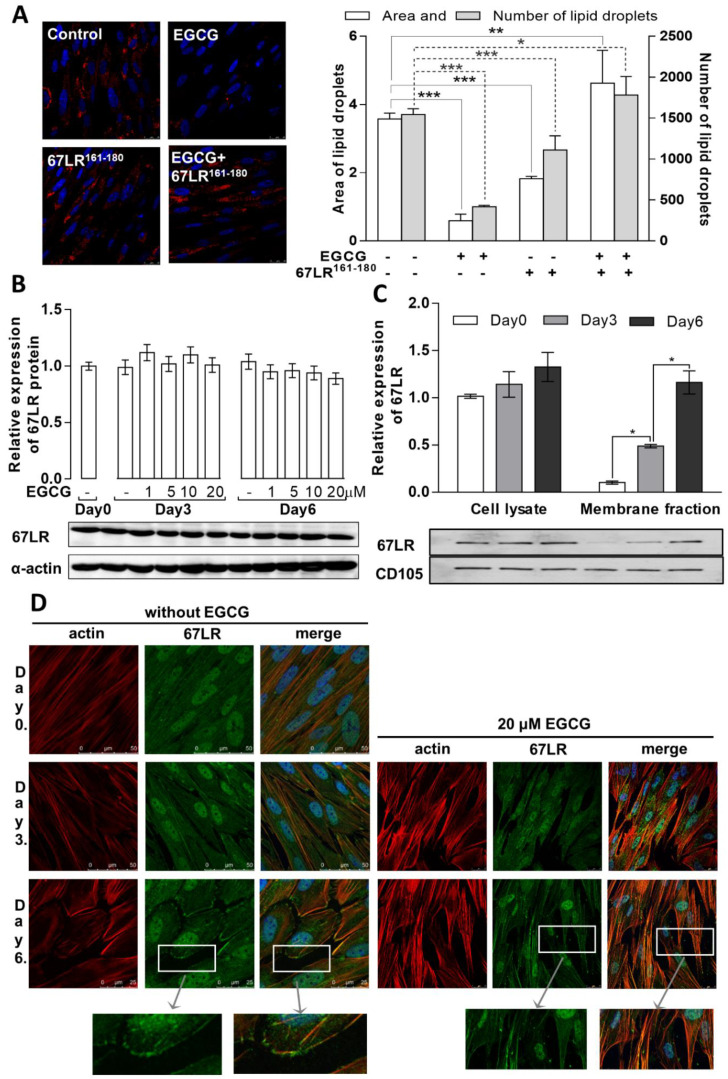
The effects of an EGCG-binding 67LR peptide (67LR^161-180^) and EGCG on lipid droplet formation, and expression and localization of 67LR in differentiating MSCs. (**A**) The effects of EGCG (20 µM), 67LR^161-180^ (20 µM), or EGCG (20 µM) plus 67LR^161-180^ (20 µM) on lipid droplet (red) formation on day 6 of the adipogenic differentiation of MSCs (left panel) and ImageJ analyses of the area and number of droplets (right panel, *n* = 5). Treatments were compared with two-way ANOVA; * *p* < 0.05, ** *p* < 0.01, *** *p* < 0.001. (**B**) MSCs were differentiated in the absence or presence of 1, 5, 10, and 20 µM EGCG. On days 0, 3, and 6, cells were lysed and subjected to Western analysis with an anti-67LR antibody. Representative Western blots are shown. Bar graphs represent densitometric analysis of the blots (*n* = 3 per group). (**C**) Analysis of 67LR in cell lysates and isolated membrane fractions of differentiating MSCs on days 0, 3, and 6 of adipogenic differentiation. Representative Western blots are shown. Bar graphs represent densitometric analysis of the blots (*n* = 3). Treatments were compared with two-way ANOVA; * *p* < 0.05. (**D**) Confocal images of differentiating MSCs in the absence and presence of 20 µM EGCG on the indicated days with nuclear staining using DAPI (blue, not shown separately), actin staining using Texas Red Phalloidin (red), and anti-67LR antibody followed by fluorescent secondary antibody (green). Scale bar: 50 μm.

The confocal images in Figure 2A (left panel) show lipid droplet formation in the absence or presence of EGCG, 67LR^161-180^, or EGCG plus 67LR^161-180^ on day 6 of differentiation. Similar to the results from Figure 1B, a significant amount of lipid droplets was identified on day 6 in the untreated samples and EGCG suppressed droplet formation, which was confirmed by the quantification of the area and the number of the droplets on the images (Figure 2A, right panel). Lipid droplet formation in the presence of 67LR^161-180^ decreased also compared to the control. In the presence of both EGCG and 67LR^161-180^, the droplet formation was restored to the control levels, suggesting that 67LR^161-180^ competes with EGCG for 67LR binding. Therefore, the suppressive effect of EGCG was reversed in the presence of the peptide.

The relationship between the expression and localization of 67LR and the effects of EGCG on the adipogenic differentiation of MSCs were investigated next. As shown in Figure 2B, the expression levels of 67LR protein did not change during adipogenesis either in the absence or presence of EGCG. Using Western blotting, 67LR was detected in cell lysates and membrane fractions after subcellular fractionation. The translocation of 67LR to the membrane fraction was detected on day 3 and became more evident on day 6 of differentiation (Figure 2C, left panel). As shown in the confocal microscopic images (Figure 2D), 67LR was predominantly localized in the nucleus and cytosol of undifferentiated MSCs (Day 0), and partial co-localization with actin filaments was apparent. The localization patterns of 67LR and actin on day 3 were similar to those on day 0, except for some colocalization to the membrane. On day 6, the membrane localization of 67LR was observed (see Figure 2D, left panel). The localization of actin beneath the cell membranes of the differentiated cells was also observed on day 6, accompanied by changes in cell shape from elongated to rounded. The localization of 67LR was also examined in the presence of EGCG on days 3 and 6 (Figure 2D, right panel). Although the shape of the cells was different in the presence of EGCG, the localization pattern of 67LR appeared to be similar to that observed in the absence of EGCG.

These results indicate that the influence of EGCG on adipogenesis requires the translocation of 67LR to the cell membrane and is exerted via EGCG–67LR interactions, as the effects of EGCG are more pronounced on day 6 when 67LR is localized to the membrane and available to bind this agonist.

### 3.3. Effects of EGCG on the Expression and Phosphorylation of Key Transcription Factors, Adiponectin, and PP2A Activity

**Figure 3:**
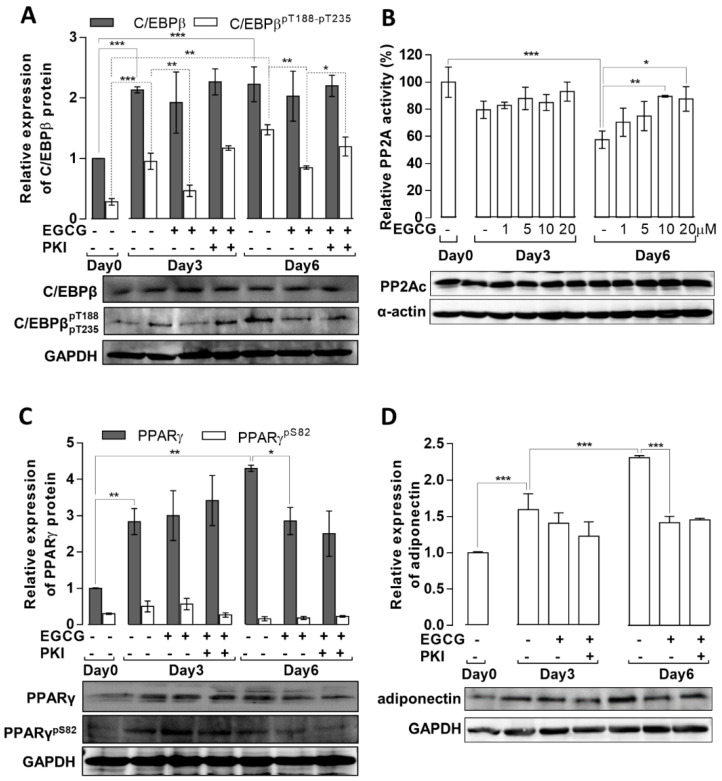
Expression and phosphorylation of C/EBPβ, PPARγ, and adiponectin, and changes in PP2A activity during the adipogenic differentiation of MSCs. MSCs were differentiated in the absence or presence of 20 μM EGCG or 20 μM EGCG plus 20 μM PKI. On days 0, 3, and 6 of differentiation, cells were lysed and subjected to Western blot analysis or phosphatase activity assays, as described in the Materials and Methods section. (**A**) Expression and phosphorylation of C/EBPβ detected with anti-C/EBPβ and anti-C/EBPβ^p188/235^. (**B**) PP2A specific activity in cell lysates measured in the presence of 2 µM PP1 inhibitor (inhibitor-2), using pMLC20 as substrate. Under the bar graph of phosphatase assay, Western blots of PP2A catalytic subunit from activity measurements are shown together with blots of α-actin, as a housekeeping protein. (**C**) Expression and phosphorylation of PPARγ were detected with anti-PPARγ and anti-PPARγ^p82/112^. (**D**) Expression of adiponectin assessed by anti-adiponectin antibody. In A, B, C, and D, representative Western blots are shown. Bar graphs represent densitometric analysis of the blots (*n* = 3). Statistical analysis: two-way ANOVA; * *p* < 0.05, ** *p* < 0.01, *** *p* < 0.001; n.s.: non-significant.

Changes in protein expression and the phosphorylation levels of C/EBPβ and PPARγ, two key transcription factors in adipocyte development, during the adipogenic differentiation of MSCs were determined. In the absence of EGCG, total C/EBPβ increased about two-fold on day 3 and remained at a similar level on day 6 (Figure 3A). EGCG did not significantly influence C/EBPβ expression. C/EBPβ phosphorylation on T188/T235 (pC/EBPβ) increased significantly on day 3 and was slightly elevated on day 6. When differentiation occurred in the presence of EGCG (20 µM), pC/EBPβ levels were significantly lower on days 3 and 6 compared with pC/EBPβ levels in the absence of EGCG.

As PP2A is involved in the dephosphorylation of pC/EBPβ [12], we determined PP2A phosphatase activity in lysates from differentiated cells in the absence or presence of EGCG, using phosphorylated 20 kDa light chain of myosin (pMLC20) as a substrate. As pMLC20 is dephosphorylated by both PP2A and PP1, the phosphatase activity due to PP2A was obtained by the specific inhibition of PP1 with inhibitor-2 protein [37]. It should be noted that other inhibitor-2 insensitive type-2 (Ca^2+^-dependent PP2B and Mg^2+^-dependent PP2C) phosphatase as well as PP2A-like PP4 and PP6 may also contribute to this phosphatase activity. All of these phosphatases include metal-ions at their catalytic centers; however, PP2B and PP2C, which also dephosphorylate pMLC20, require extra Ca^2+^ or Mg^2+^, respectively, for phosphatase activity. In our experiments, the phosphatase assays of cell lysates were carried out in the presence of EDTA; therefore, PP2B and PP2C were not active under these conditions as it was reported earlier [41]. It has not been shown if PP4 or PP6 dephosphorylates pMLC20 or not, but a previous report [42] indicated that PP4 and PP6 dephosphorylated PP2A substrates (phosphorylase-*a* and phospho-histonH1) 84–140-fold less efficiently than did PP2A. The above data support our view that the predominant phosphatase activity we determined in our assays in the presence of inhibitor-2 is due to PP2A, which might be applicable to pMLC20, too. As shown in Figure 3B, PP2A activity did not change significantly on day 3 compared to the control (day 0). In contrast, PP2A activity was significantly reduced on day 6 in the absence of EGCG. This decreased PP2A activity was enhanced by EGCG in a concentration-dependent manner on day 6 of differentiation. The expression of the PP2A catalytic subunit (PP2Ac) did not change significantly during the course of differentiation and treatments (Figure 3B, lower panel).

The effects of EGCG on the expression and phosphorylation of C/EBPβ were also probed in the presence of a PKA inhibitor (PKI) to determine if EGCG exerted its effects via the EGCG–67LR→cAMP→PKA pathway with the consequent activation of PP2A [31].

PKI did not influence the expression of C/EBPβ in the presence of EGCG (Figure 3A). However, PKI increased pC/EBPβ in the presence of EGCG on days 3 and 6 to the levels observed in the absence of EGCG. These results indicate that EGCG induces the dephosphorylation of pC/EBPβ via the activation of PP2A in a PKA-dependent manner. These data also confirm previous results that implicated the Bδ-subunit-associated PP2A in the dephosphorylation of pC/EBPβ [12]. Earlier studies showed that PP2A–Bδ was activated by the phosphorylation of Bδ by PKA [32].

In the absence of EGCG, PPARγ expression increased on days 3 and 6. The amount of phosphorylated PPARγ rose slightly on day 3 and decreased on day 6 compared to the control; however, neither of these changes were significant (Figure 3C). In the presence of EGCG, PPARγ expression was partially suppressed on day 6. Neither PPARγ expression nor phosphorylation was influenced by PKI. Changes in another adipogenic factor, adiponectin, were also assayed during the differentiation of MSCs (Figure 3D). In the absence of EGCG, the expression of adiponectin was elevated on days 3 and 6 of MSCs differentiation. EGCG partially suppressed the expression of adiponectin on days 3 and 6 independent of PKI (Figure 3D).

The above data imply that the suppression of adipogenesis by EGCG, at least in part, may be due to the partial inactivation of pC/EBPβ by dephosphorylation together with the suppression of PPARγ expression, resulting in the negative regulation of the expression of adipogenic genes, such as adiponectin.

### 3.4. Effects of EGCG on the MSC Cytoskeletal Rearrangement and Phosphorylation of MLC20 during Adipogenesis

**Figure 4:**
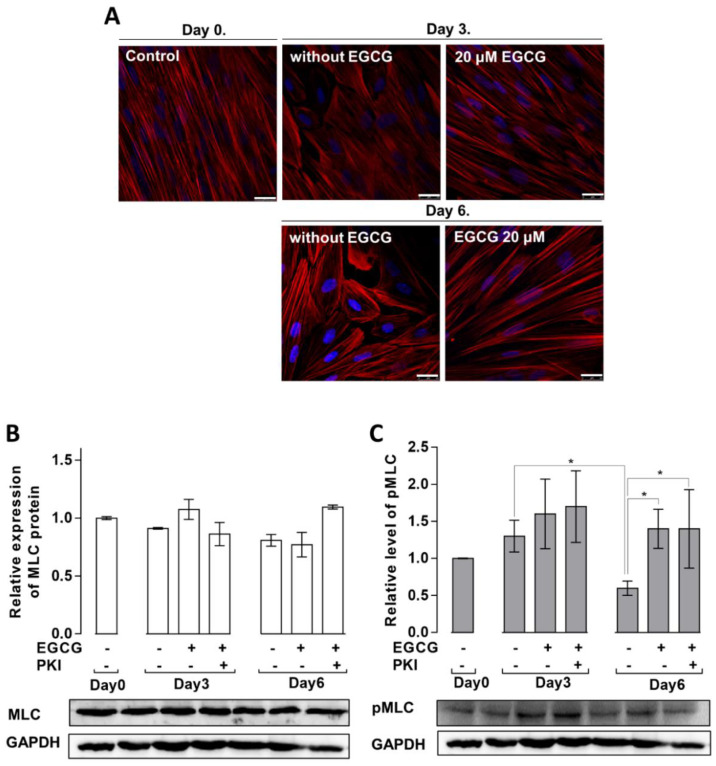
Changes in actin cytoskeleton and myosin phosphorylation in MSCs differentiated in the absence or presence of EGCG. (**A**) Confocal images of actin staining with Texas Red Phalloidin (red) on the indicated days of differentiation in the absence or presence of EGCG. Scale bar: 25 μm. Expression (**B**) and phosphorylation (**C**) of MLC20 during the adipogenic differentiation of MSCs in the absence or presence of EGCG or EGCG plus PKI. Representative Western blots are shown. Bar graphs represent the densitometric analysis of the blots (*n* = 3). Statistical analysis: two-way ANOVA; * *p* < 0.05.

Upon the induction of differentiation, the rearrangement of the actin cytoskeleton may determine the fate of MSCs to osteoblasts or adipocytes [16,17]. Undifferentiated MSCs (day 0) exhibited actin–myosin stress fibers with elongated and flattened shapes and this pattern changed slightly on day 3 in the absence, but not in the presence of EGCG (Figure 4A). The amount of stress fibers decreased by day 6 of differentiation, and the cells became rounded, in part due to the rearrangement of the actin cytoskeleton, forming mainly cortical actin filaments and also to the accumulation of lipid droplets in the cells (see Figure 1B). In contrast, the amount of stress fibers and the cell shape were unchanged on day 6 of differentiation after EGCG treatment (20 μM) compared to days 0 or 3 (Figure 4A).

The appearance of stress fibers generally requires the phosphorylation of non-muscle MLC20. Therefore, the expression (Figure 4B) and phosphorylation (Figure 4C) of MLC20 protein during MSC differentiation were determined.

As shown in Figure 4B, no significant changes in the expression of MLC20 were detected during the differentiation of MSCs, either in the absence or presence of EGCG or EGCG plus PKI. The amount of phosphorylated MLC20 (pMLC20) on day 3 was similar to control (day 0) in the absence and presence of EGCG or EGCG plus PKI. In contrast, on day 6, pMLC20 was significantly reduced in the absence of EGCG. This reduction may contribute to the observed disassembly of stress fibers allowing the cell shape to become rounded, which is advantageous for the accumulation of lipid droplets (Figure 4C). In the presence of EGCG or EGCG plus PKI, pMLC20 remained phosphorylated in accord with the sustained stress fibers and elongated shapes of cells observed on confocal images (Figure 4A).

**Figure 5:**
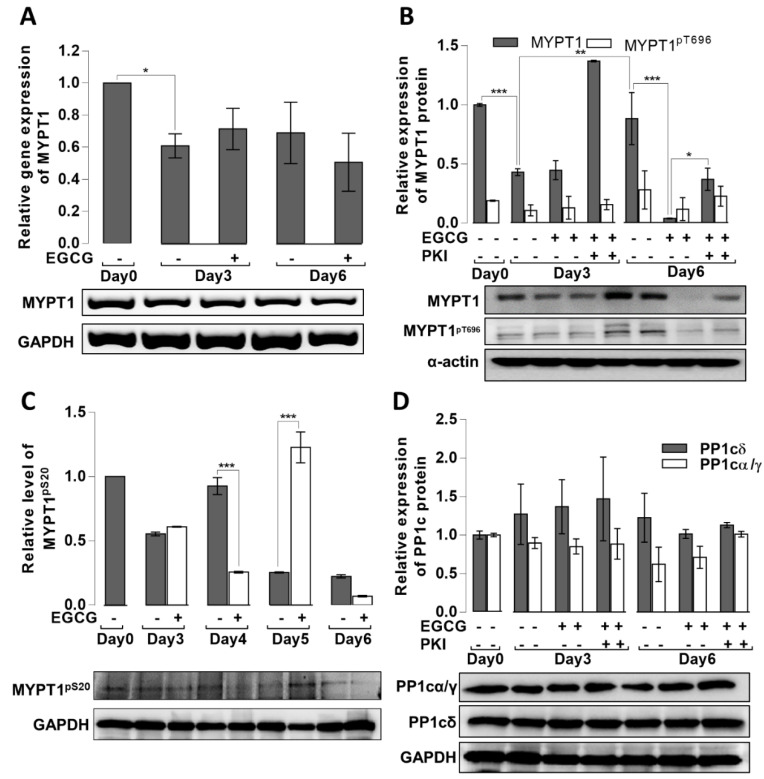
Expression and phosphorylation of MYPT1 and PP1c isoforms during the adipogenic differentiation of MSCs. MSCs were differentiated in the absence or presence of EGCG (20 µM) or EGCG plus PKI (20 µM). (**A**) Expression of the MYPT1 gene was assessed, as described in the Materials and Methods section. (**B**) MYPT1 protein levels and phosphorylation at Thr696 were determined by Western blots using anti-MYPT1^1-296^ and anti-MYPT1^pThr696^ antibodies. (**C**) Phosphorylation of MYPT1 at Ser20 determined by Western blots using an anti-MYPT1pSer20 antibody. (**D**) Assessment of protein expression of PP1c isoforms using PP1cδ and PP1cα/γ1 specific antibodies. Representative blots are shown. Bar graphs represent densitometric analysis of the blots (*n* = 3). Statistical analysis: two-way ANOVA: * *p* < 0.05, ** *p* < 0.01, *** *p* < 0.001, n.s.: non-significant.

The results showing that EGCG increases rather than decreases pMLC20 are quite intriguing, as numerous studies have demonstrated that EGCG induces the activation of MP and the dephosphorylation of pMLC20 [33,34,35]. To uncover the possible mechanisms behind these unexpected events, we assessed MYPT1 gene and protein levels in the absence or presence of EGCG during the adipogenic differentiation of MSCs. As shown in Figure 5A, MYPT1 gene expression decreased significantly by day 3 (~40% decrease) independent of EGCG. The MYPT1 protein also decreased (~60% decrease) on day 3 of differentiation independent of EGCG; however, in the presence of EGCG plus PKI, it was increased (Figure 5B). The MYPT1 protein level significantly increased on day 6 compared to those on day 3 in the absence of EGCG. In contrast, in the presence of EGCG, MYPT1 almost disappeared from the cells; this effect was only partially reversed by PKI. These data support the conclusion that EGCG induces the degradation of MYPT1 and the inhibition of PKA might protect MYPT1, at least in part, from this proteolytic effect. A previous study showed that the phosphorylation of MYPT1 at Ser20 may sensitize this protein to ubiquitination and subsequent degradation [43]. Figure 5C shows that the phosphorylation of MYPT1 at Ser20 was predominant on day 5 of differentiation, which might prepare MYPT1 for ubiquitination-mediated degradation.

MYPT1 has an essential role in targeting PP1c to the substrate myosin and myosin light chain. Therefore, losing MYPT1 may cause the less effective dephosphorylation of pMLC20 and may explain the increased level of pMLC20 in the EGCG-treated cells. For increased cellular pMLC20 levels, other alternatives are the inhibitory phosphorylation of MYPT1 and/or the decreased expression of PP1c. Figure 5B shows that the inhibitory phosphorylation of MYPT1 at the assessed Thr696 was low and did not change significantly during the course of adipogenic differentiation. Changes in the expression of distinct PP1c isoforms were determined by Western blots using antibodies specific for PP1cδ and both PP1α and PP1γ1, but not PP1cδ [37]. As shown in Figure 5D, the expression of PP1c isoforms changed during adipogenesis; however, the changes were not significant.

Overall, the results are in accordance with the presumption that the rise in pMLC20 on day 6 of differentiation in the presence of EGCG is due to the proteolytic degradation of MYPT1 coupled with its decreased targeting function toward the pMLC20 substrate.

## 4. Discussion

EGCG is the major polyphenol in green tea and appears to be responsible for numerous beneficial cellular effects. Previous studies established the influence of EGCG on the differentiation of pre-adipocytes or MSCs to adipocytes. The majority of these reports showed that EGCG suppressed adipogenic differentiation [21,25,26,44]; however, the opposite findings for EGCG and related catechins were also demonstrated [20]. Our present results are consistent with the conclusion that EGCG is an inhibitor of the adipogenic differentiation of MSCs and show a possible mechanism for this process. Many intracellular effects of EGCG are exerted via its action as an agonist of 67LR [30]. The binding of EGCG to 67LR initiates different pathways and leads to the activation of distinct protein kinases and phosphatases, including PKA and PP2A [31], MP [33], PKG, and PKCδ [45]. Our results suggest that EGCG may suppress the adipogenesis of MSCs through its interaction with 67LR. The following experimental findings support this conclusion: (i) the action of EGCG is counteracted by a peptide representing the EGCG-binding region of 67LR (Figure 2A), and (ii) the EGCG suppression of adipogenesis is most prominent on day 6 of MSC differentiation (see Figure 1), when 67LR translocation to the cell membrane is predominant (Figure 2B,C). The reasons for the differentiation-dependent translocation of 67LR to the plasma membrane are not known. Previous studies presumed that the cytoplasmic 37LR precursor homo- or heterodimerized [46] and became fatty acylated [47] to form the membrane-bound 67LR. However, the precise mechanisms for these events have not been established. Nevertheless, our data imply that the translocation and agonist engagement of 67LR may be an important factor influencing the differentiation of MSCs, presumably in not only adipogenesis, but other cellular processes as well.

The above mechanisms may also explain the stimulated dephosphorylation of pC/EBPβ, as EGCG activates protein phosphatases via the EGCG–67LR→cAMP→PKA→PP2A signaling pathway [31], leading to enhanced PKA-dependent PP2A activity (Figure 3B). The novel aspects of the present study include the determination of PP2A-specific activity in differentiating MSCs and the demonstration of EGCG effects on day 6 of differentiation when the above effects of EGCG were predominant. To date, the only PP2A holoenzyme that is activated by phosphorylation via PKA is the B56δ subunit of the ABC heterotrimer [32]. The PP2A heterotrimer, including the B56δ subunit, dephosphorylates pC/EBPβ in 3T3-L1 preadipocytes [12]. Thus, our present results direct attention toward the regulation of adipogenic MSC differentiation by EGCG via signaling pathways that include changes in the activity of protein kinases and phosphatases such as PKA and PP2A.

Our data imply that EGCG induces the dephosphorylation of a phosphorylation site (pThr188/235) in pC/EBPβ, which increases the transcriptional activity of this protein (Figure 3A,B) and partially explains the suppressive effect on the adipogenesis of MSCs. However, in light of previous studies, the interpretation of our present results is not without controversy, including the timing of C/EBPβ expression and phosphorylation during the differentiation processes. Using 3T3-L1 pre-adipocytes, the expression and phosphorylation of C/EBPβ varied widely during the time course of adipogenic differentiation. C/EBPβ was transiently elevated in the initial phase of differentiation and declined by the end of day 1 [48]. Similar transient changes were detected for the levels of pC/EBPβ in the first 24 h of differentiation, while C/EBPβ protein remained high up to day 2 [49]. Other reports identified the significant expression of C/EBPβ during days 2 to 7 of differentiation [50,51], and pC/EBPβ was still detected on day 2 [50]. In MSCs, C/EBPβ gene expression increased through day 14 [52] and C/EBPβ protein was present at significant amounts from days 2 to 8 during differentiation [53]. However, less is known about the presence and lifetime of pC/EBPβ. Our present findings indicate that the phosphorylation of C/EBPβ at Thr188/235 increased on days 3 and 6 during the later phase of MSC differentiation. As this phosphorylation enhances the transcriptional activity of C/EBPβ, we can assume that the EGCG-induced PKA-dependent dephosphorylation of pC/EBPβ by PP2A is coupled with the partial suppression of adipogenic differentiation. EGCG also suppressed the expression of PPARγ (Figure 3C) and adiponectin (Figure 3D) on day 6 of differentiation, and these alterations may contribute to its suppressive effects on adipogenesis. These influences occurred in a PKA-independent manner, implying that C/EBPβ regulates the expression of both PPARγ and adiponectin, but its phosphorylation may not be involved in these events.

In addition to PP2A, PP1 also has regulatory roles in adipogenesis, and PP1cδ is required for the promotion of adipogenesis of 3T3-L1 cells [11]. PP1c does not act alone as a free catalytic subunit, but associates with distinct regulatory subunits to form functional holoenzymes. MP is a well-known holoenzyme in which PP1cδ is specifically associated with the MYPT1 targeting subunit. The major substrate for MP is MLC20. Therefore, the physiological roles of MP in smooth muscle and cell contractility have been investigated in great detail [18]. However, MP may also have an important impact on other cellular processes [19]. The possibility that the role of MP in adipogenesis is influenced by EGCG is supported by previous findings, as follows: (i) MP could be activated via the signaling pathway of EGCG–67LR→cAMP→PKA→PP2A→MP initiated by the EGCG–67LR interaction [33,34,35], and (ii) the fate of MSCs to differentiate in distinct directions (e.g., adipogenesis or osteogenesis) is determined by the shape and stiffness of MSCs [15,16,17]. The latter conditions are regulated via myosin phosphorylation, which is mediated by MP. In agreement with previous studies, our results show that adipogenesis is promoted by a rounded cell shape coupled with the low-level phosphorylation of MLC20. Therefore, the expectation that EGCG, which activates MP in other cell types, promotes the development of a rounded cell shape and the dephosphorylation of MLC20 is reasonable. However, rather surprisingly, our experiments identified the opposite effects. In the presence of EGCG, the elongated shape of cells with stress fibers was sustained with increased MLC20 phosphorylation (see Figure 4). Our results show that MYPT1 was almost completely degraded upon EGCG treatment in cells on day 6 of differentiation. This implies that the targeting role of MYPT1 (directing PP1c toward the substrate) is lost, which is reflected in the decreased dephosphorylation of MLC20 and increased stress fiber formation and stiffness of cells. These changes in cell shape do not favor lipid accumulation, contributing to the suppression of adipogenesis.

The question arises of which EGCG-dependent mechanism initiates the degradation of MYPT1 and possibly other proteins. Proteolytic cleavage and the degradation of MYPT1 have been previously reported [54,55]. MYPT1 was cleaved by caspase-3 in apoptotic HeLa cells at the C-terminal region (D884); in this case, MYPT1 remained phosphorylatable at the inhibitory sites (Thr696 and Thr853), but lost its binding to myosin [54]. The molecular interaction between MYPT1 and the E3-ligase SIAH2 resulted in the targeting of MYPT1 to the ubiquitin-proteasome pathway for degradation [55]. The phosphorylation of MYPT1 at Ser20 by Chk1 kinase sensitized the protein to ubiquitination and subsequent proteasomal degradation [43]. The use of caspase-3 or proteasomal inhibitors may help to determine the mechanism of MYPT1 degradation; however, their application to MSCs during the course of differentiation caused cell death before the experiments could be completed (results are not shown). It is unlikely that MYPT1 was cleaved by caspase-3 in our model, as no lower molecular mass MYPT1 fragment was identified on Western blots nor was phosphorylation detected at the inhibitory sites (see Figure 5). On the other hand, the increased phosphorylation of MYPT1 at Ser20 is apparent on day 5 and this event may be responsible for sensitizing MYPT1 to ubiquitination and proteasomal degradation on day 6. The EGCG induction of protein degradation has been demonstrated in previous studies; EGCG mediated the protein kinase C- and proteasome-dependent degradation of Bad [56] in neuronal cells and prevented the aggregation of pulmonary-fibrosis-associated mutant surfactant protein A2 via a proteasomal degradation pathway [57]. Moreover, cAMP signaling may initiate proteasomal degradation pathways in both PKA-dependent [58,59], PKA-independent, and EPAC1-dependent manners [60]. Thus, EGCG-induced cAMP and PKA activities via EGCG–67LR interactions could initiate the proteasomal degradation of proteins, and, at least in part, this mechanism may mediate MYPT1 degradation in MSCs. The involvement of PKA in these processes is evident from our data showing that the inhibition of PKA partially rescues MYPT1 (see Figure 5) from degradation.

## 5. Conclusions

Our present data highlight the roles of two specific PP1 and PP2A holoenzymes, PP2A-ABδC and MP (PP1cδ-MYPT1), in the signaling of adipogenesis in MSCs by EGCG. EGCG suppresses adipogenesis via binding to 67LR, which is translocated to the cell membrane during differentiation; EGCG–67LR interactions at the cell membrane lead to the activation of PP2A and degradation of the MYPT1 subunit of MP, with a parallel decrease in the phosphatase activity toward phosphorylated myosin. These latter events represent a novel mechanism regulating the phosphorylation levels of MP substrates; however, to clarify the mechanisms of MYPT1 downregulation, further research is needed. PP2A mediates the phosphorylation of key transcription factors, C/EBPβ and PPARγ, while MP modulates cell shapes via actin cytoskeleton rearrangements. Overall, our present study directs closer attention to the regulation of adipogenesis via intracellular pathways initiated by the agonist-receptor interaction of EGCG with 67LR and the consequent changes in the activity of different P-Ser/Thr specific protein phosphatases.
